# MicroRNAs: understanding their role in gene expression and cancer

**DOI:** 10.31744/einstein_journal/2021RB5996

**Published:** 2021-07-08

**Authors:** Ariany Lima Jorge, Erik Ribeiro Pereira, Christian Sousa de Oliveira, Eduardo dos Santos Ferreira, Edmara Toledo Ninzoli Menon, Susana Nogueira Diniz, Julia Alejandra Pezuk

**Affiliations:** 1 Universidade Anhanguera de São Paulo São PauloSP Brazil Universidade Anhanguera de São Paulo, São Paulo, SP, Brazil.

**Keywords:** MicroRNAs, Gene expression, Neoplasms, Biomarkers

## Abstract

MicroRNAs are small RNA molecules that regulate gene expression in cells. These small molecules comprise 17 to 25 nucleotides and are able to recognize target messenger RNAs by sequence complementarity and regulate their protein translation. Different microRNAs are expressed in all human cells. There is over 2,500 microRNAs described in humans that are involved in virtually all biological processes. Given their role as gene expression regulators, these molecules have been widely investigated and are thought to be associated with some specific physiological and pathological conditions, being proposed as biomarkers. It has recently been reported that microRNAs are secreted outside cells and are involved in intercellular communication. MicroRNAs in biological fluids are named circulating and have been detected in all body fluids, although the expression profile is specific for each type. The major advantages of using circulating microRNAs as biological markers are the high stability of those molecules and the wide availability of samples. Also, given the individual nature of microRNA expression changes, these molecules have a high potential for use in personalized medicine. In fact, microRNA expression profile determination may support disease recognition and diagnosis, and can be used to monitor therapeutic responses and establish patient prognosis, assisting in choice of treatment. This review provides a general overview of microRNAs and discusses the importance of those molecules in cancer, for deeper understanding of their role in this disease.

## INTRODUCTION

MicroRNAs (miRNAs) are small non-coding ribonucleic acid (RNA) molecules that do not encode proteins. These molecules have drawn attention in the last few years due their role in gene expression regulation and involvement in diseases, such as cancer. The term cancer refers to a group of genetic diseases that may be caused by inactivation of tumor suppressor genes and/or activation of proto-oncogenes. Cell malignancy is associated with accumulation of deoxyribonucleic acid (DNA) mutations, which lead to gene expression dysregulation, resulting in biological and functional effects on cells. Epigenetic processes have also been incriminated in malignant tumor development. Among them miRNAs have gained visibility due to their relation with the acquisition of malignant characteristics during carcinogenesis.^(^^1^^)^

This review discusses the latest scientific data on miRNA generation and function, as well as their relations with cancer. Updated information about the role of these molecules is presented and their potential applicability in cancer treatment and monitoring is discussed.

## MicroRNAS

MicroRNAs were first described in *Caenorhabditis elegans* in the 1990´s, and are thought to be vital for gene expression regulation in animal and plant cells.^(^^2^^)^ These molecules comprise 17 to 25 nucleotides and regulate gene expression at the post-translational level, leading to changes in the pattern of protein translation via interactions with messenger RNAs (mRNAs).^(^^3^^)^ The miRBase database ( www.mirbase.org ) lists more than 18 thousand different miRNAs in 168 species. Over 2,500 miRNAs have been identified in humans. However, the function of many of them has not been completely understood.^(^^4^^)^ Variations in miRNA expression patterns are thought to be related to several biological and physiological processes and have been described in almost all diseases in humans.^(^^5^^,^^6^^)^

## MicroRNA BIOGENESIS

**Figure 1:**
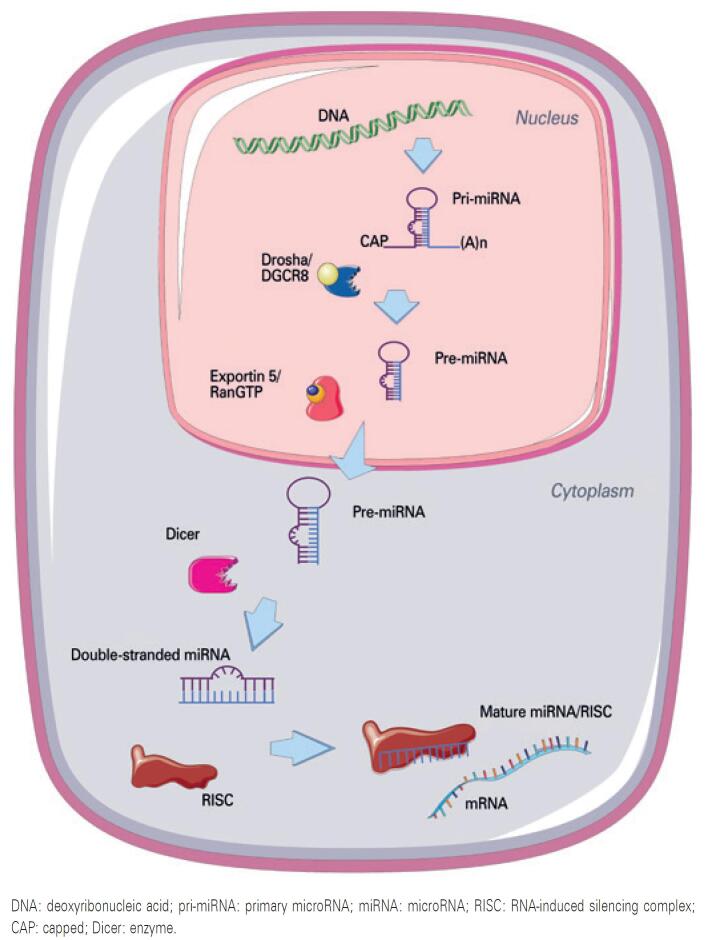
Canonical microRNA biogenesis pathway. This pathway begins with nuclear transcription of microRNAs into molecules named pri-microRNAs. Pri-microRNAs are then processed by the nuclear enzyme Drosha to generate smaller molecules, the so-called pre-microRNAs. Pre-microRNAs are exported to the cytoplasm by exportin-5, then processed by the enzyme Dicer, giving rise to double-stranded microRNA molecules, which bind to RNA-induced silencing complex. The strands of bound microRNAs are then separated, originating to mature microRNAs, which are able to bind to messenger RNAs and inhibit their translation

MicroRNA generation is a complex process, which starts in the nucleus and ends in the cytoplasm. This process involves several enzymes and cell protein complexes, which regulate the pathway that leads to the production of mature and functional miRNAs ( Figure 1 ). At least three miRNA production pathways have been described, the canonical being the most widely investigated.^(^^3^^)^

The canonical pathway of biogenesis begins with DNA transcription, and the miRNAs may be encoded in intragenic (particularly intronic) or intergenic regions, both of the sense or antisense DNA strand. The transcription is often mediated by RNA polymerase II or RNA polymerase III.^(^^7^^)^

The first transcript (primary miRNA or pri-miRNA) has a hairpin structure. This structure has a base-paired stem that forms a double-stranded RNA region (dsRNA), protected by a capped (CAP) and a poly-A tail at the ends.^(^^8^^)^ The primary miRNA is processed in the nucleus by an enzyme complex formed by RNase III Drosha and the protein Pasha or DGCR8 (DiGeorge Syndrome Critical Region 8), which binds to the double stranded RNA molecule, leading to uncapping and poly-A tail removal. This results in a double-stranded RNA molecule comprising approximately 70 nucleotides, the precursor miRNA (pre-miRNA), which is exported to the cytoplasm by exportin-5 and Ran-GTP binding proteins.^(^^9^^)^ In the cytoplasm, the miRNA is further processed by the Dicer enzyme and the not base-paired loop of the molecule is removed, originating a double-stranded miRNA containing approximately 22 nucleotides. Alternatively, Argonaut 2 protein (Ago2) may be involved in pre-miRNA cleavage in the cytoplasm.^(^^10^^)^ This double stranded miRNA is associated with a protein complex known as RNA-induced silencing complex (RISC), which is formed by several proteins. Ago proteins are among the most important factors in RISC, given their key role in the interaction with RNA molecules.^(^^11^^)^ RNA-induced silencing complex leads to miRNA strand separation. One strand is often degraded, whereas the other, actually the mature miRNA, is exposed and may pair with the target mRNA.^(^^3^^,^^8^^)^

In the non-canonical pathway of miRNA biogenesis, pre-miRNA production takes place in the nucleus, starting from other molecules, such as shRNA (short hairpin RNA), miRtron or m7G-pre-miRN, and some subsequent steps may vary. Pre-miRNA generation from miRtron involves the spliceosome within the nucleus, whereas transportation of m7G-pre-miRN to the cytoplasm is exportin-1-dependent. Finally, the difference between canonical and non-canonical processing involving shRNAs, its due to the participation of Ago2 proteins instead of Dicer.^(^^3^^,^^12^^)^

## MECHANISM OF ACTION OF MicroRNAs

MiRNA binds to target mRNAs through sequence complementarity, leading to changes in the translation pattern of mRNA proteins. Pairing may be total or partial and, apparently, the greater the sequence complementarity, the stronger and longer lasting the interaction. MiRNAs are thought to be important regulators of gene expression and act via dynamic mechanisms which are influenced by molecule localization and mechanisms of interaction with targets.^(^^3^^)^

The mechanism of action of miRNAs includes several steps, and involves several proteins.^(^^12^^)^ MiRNAs bind to their targets primarily at untranslated region 3’ (UTR). Alternatively, miRNAs bind to other regions, such as 5’UTR, or even to promoter regions in the target mRNA. Binding site determines the biological consequences of these interactions. Most miRNAs inhibit gene expression by hindering target mRNA protein translation. However, the binding of a miRNA to the promoter region of a gene may have the opposite effect, that is, enhanced gene expression.^(^^13^^)^

Interactions between miRNAs and target mRNAs may lead to mRNA degradation through cleavage, mediated by an endonuclease in RISC, to which the miRNA is bound.^(^^14^^)^ At first, total complementarity was thought to be required for this step, and only transient inhibition was thought possible in cases of partial complementarity. However, more recent studies have shown that, in animal cells, most miRNAs are only partially complementary to target mRNAs, which seems to be enough to permanently inhibit target mRNA translation.^(^^5^^,^^15^^)^

The small size of miRNAs and the tolerability to partial complementarity with its targets allows interactions with many mRNAs. The biological consequences of variations in a given miRNA may differ according to the cellular context. Hence, experimental verification is required, considering the cellular effects of miRNAs depend on target mRNA function. In the presence of sequence complementarity with target mRNAs with antagonistic functions, a single miRNA may have opposite biological effects in different cells. Therefore, to understand the role of different miRNAs, the profile of mRNAs transcripts in each cell type must be taken into account. Given the complexity of miRNA-mRNA interaction networks, bioinformatics modeling may be used for deeper understanding of this relation and recognition of additive or contradictory effects of different miRNAs.^(^^3^^)^

Also, each mRNA can be the target of more than one miRNA. To understand the effects of a given miRNA, interactions between the target mRNA and other miRNAs must be considered, since biological outcomes may reflect the sum of effects of several miRNAs expressed in the same cell.^(^^4^^)^ Therefore, it can be argued that a given miRNA can regulate opposite processes in different cell types, such as increased cell proliferation and apoptosis rates. Nonetheless, similar levels of a specific miRNA may have different biological effects, since these depend upon targets and interactions with other molecules in a given cell type.

## MicroRNAS IN CANCER

The role of miRNAs in cancer has been the object of investigations since the early 2000´s, when Calin et al.,^(^^16^^)^ reported decreased expression of miR-15 and miR-16 in patients with chronic lymphoblastic leukemia. Ever since, important relations between the dysregulation of these molecules and carcinogenesis have been proposed, based on changes in expression of several miRNAs in tumor cells.^(^^17^^,^^18^^)^ In theory, all tumors have some level of dysregulated miRNA expression. Recent data suggest more than half of miRNA codifying genes in humans are located in genomic regions that have been shown to be dysregulated in cancer.^(^^4^^,^^19^^)^

Cancer-related miRNAs can be classified according to target mRNA function as tumor suppressor or oncogenic miRNAs (oncomiRs). This segregation is based on the ability of miRNA molecules to interfere in carcinogenesis-related processes, including mechanisms associated with cell migration and invasion, apoptosis and proliferation. Given most miRNAs inhibit target mRNA expression, miRNA and target RNA may have opposite classifications. Tumor suppressor miRNAs regulate the expression of mRNAs required for cell division or survival, whereas oncomiRs are more strongly expressed in cancer cells and down-regulate tumor suppressor genes, leading to enhanced cancer cell division.^(^^1^^)^

Changes in miRNA expression patterns detected in cancer cells emphasize the significant role of miRNAs in cancer development and progression.^(^^20^^)^ Variations in miRNA profile have been associated with processes driving cancer progression, metastasis formation and even cell death rates, and may be related to patient prognosis. Dysregulated miRNA expression in some malignant tumors that progress or metastasize justifies the investigation of these molecules as potential therapeutic targets in advanced cancer.^(^^21^^)^ In addition, defining microRNA expression profile may be useful to identify and diagnose tumor subtypes, and to revise prognosis in oncologic patients.^(^^17^^,^^18^^)^

There has recently been an exponential growth in the number of studies investigating relations between miRNAs and cancer. Literature searches conducted at Pubmed^®^ ( www.pubmed.ncbi.nih.gov ) and Scientific Electronic Library Online (Scielo) ( www.scielo.org ) showed increasing numbers of miRNA-related scientific investigations in the last 7 years ( Table 1 ).

**Table 1:** Number of articles investigating relations between microRNAs and cancer published in the last 7 years, according to keywords cited in the title or abstract

Database	Keywords	2013	2014	2015	2016	2017	2018	2019	Total
PubMed^®^	“miRNA” and “cancer”	1,387	1,680	1,988	1,980	2,123	2,454	2,737	14,349
	“microRNA” and “cancer”	1,579	1,976	2,361	2,402	2,775	2,949	3,222	17,264
	“circulating microRNA” and “cancer”	24	32	33	32	35	35	49	240
	“circulating microRNA” and “cancer”	19	26	40	37	35	32	37	226
SciELO	“miRNA and “cancer”	4	2	1	4	4	4	3	22
	“microRNA and “cancer”	2	0	1	2	6	4	2	17
	“circulating microRNA” and “cancer”	0	0	1	0	0	0	0	1
	“circulating microRNA” and “cancer”	0	0	1	0	0	0	0	1

Understanding the particularities of different miRNAs is crucial to determine the role of these molecules in diseases and cancer. Several databases collect and provide miRNA-specific data. With the rising volume of scientific data, several databases are being created to document the characteristics of these molecules. The website tools4miRs ( www.tools4mirs.org ) lists several webpages that collect miRNA data, in which sequences, target-miRNAs and scientific publications addressing different miRNAs can be found.

One of the most popular is the miRBase, in which the properties of each miRNA, including current and prior names, related publications, and molecular sequences can be found. Links to several other databases containing complementary information about different miRNAs are also provided.^(^^4^^)^

Complete descriptions of the level of expression of miRNAs per type of tissue or cell line can be found in the miRmine website ( https://guanfiles.dcmb.med.umich.edu/mirmine/ ) and facilitate the investigation of pathological changes that may be explored as potential biomarkers. Moreover, information about cancer cell-specific miRNA expression may be found in the database miRCancer ( http://mircancer.ecu.edu/ ), where scientific data on levels of expression according to cancer type are available.

Websites and software, miRDB ( www.mirdb.org ) and miRTar ( https://mcube.nju.edu.cn/jwang/mirTar/docs/mirTar/ ), in particular, can also be used to examine the biological effects of the expression of a given miRNA. These include mathematical algorithms and complementarity analyses of molecular sequences that can be used to predict potential target mRNAs for different mature miRNAs. Given the use of specific algorithms, findings may vary across websites. Hence the need for a critical analysis. In addition, complementary data may be found in websites providing exclusively scientifically validated data for each miRNA, the most popular being miRTarBase ( https://mirtarbase.cuhk.edu.cn/∼miRTarBase/miRTarBase_2019/php/index.php ) and TarBase ( https://carolina.imis.athena-innovation.gr ).

Most data available in these websites and software are periodically updated. Novel databases are constantly being created and reformulated to include updated information derived from published, acquired miRNA knowledge.

## CIRCULATING MicroRNAs

It has recently been shown that miRNAs are not found only inside cells. These molecules are also released outside cells and can be found in several body fluids, and are called circulating miRNAs (c-miRNAs).^(^^20^^)^ C-miRNAs appear to play an important role in intercellular communication, as well as in physiological and pathological processes. C-miRNA transport and release mechanisms involve binding to proteins and/or encapsulation in lipoprotein vesicles, which protect them from enzymatic degradation. However, mechanisms responsible for c-miRNA release have not been fully understood yet.^(^^22^^,^^23^^)^

The first publication reporting the detection of c-miRNAs dates from 2008, when the presence of different amounts of miR-155 and miR-21 in the serum of healthy individuals and B-cell lymphoma patients was described.^(^^24^^)^ Within a short period of time, the presence of different levels of c-miRNAs has been demonstrated in other body fluids, including seminal fluid, colostrum, urine, saliva, breast milk, tears, amniotic fluid, bronchial secretion, plasma, pleural, peritoneal and cerebrospinal fluids.^(^^22^^)^ The presence of c-miRNAs in several biological fluids allows for horizontal transfer of these molecules between different tissues. Hence, these molecules may have pleiotropic properties, for they may trigger short and distant biological effects.

Mechanisms involved in c-miRNA selection and release are controlled and involve a specific profile for each type of biological fluid. Different cellular changes lead to particular variations, which can be explored as biological markers of physiological or pathological conditions.^(^^3^^)^ The use of c-miRNA profile in blood, plasma, serum or other body fluids to identify or monitor disease progression and therapeutic responses has been suggested.^(^^22^^)^ The major advantages of using c-miRNAs are high specificity and sensitivity, wide availability of samples and high stability. These characteristics support their handling and exploration as important tools in individualized medicine. However, the lack of standardized protocols limits the use of c-miRNA in clinical practice, mainly due to the lack of universal normalizers that enable appropriate quantification and reliable comparison of findings.^(^^21^^,^^25^^,^^26^^)^

### Circulating miRNAs in cancer

Biological cell changes observed in carcinogenesis may lead to specific variations in c-miRNA expression profile. However, changes in intracellular miRNA expression are not always correlated with c-miRNA expression in biological fluids. To understand this relation, the effects of tumor growth on the body must be taken into account. Changes in c-miRNA profile may reflect enhanced or altered expression in cancer cells. Nevertheless, expression profile changes may also occur in response to immune reactions, inflammatory processes, therapy and physiological changes.^(^^18^^,^^21^^)^

In the last few years, the use of c-miRNA profile determination to detect, predict and monitor the progression of several types of cancer has been proposed.^(^^21^^)^ The recognition of variations in c-miRNA expression profile is promising, particularly due to the benefits of these molecules for tumor classification.^(^^27^^,^^28^^)^ Moreover, variations in c-miRNA expression profile allow early identification of therapeutic responses and may be used to predict clinical outcomes and to infer survival rates.^(^^29^^)^ Given the role of c-miRNAs in intercellular communication, these molecules may affect gene expression in distant cells. Hence their important role in tumor progression and metastasis formation.^(^^29^^)^ Circulating miRNAs are thought to participate in the preparation of metastatic niches, facilitating the colonization of novel environments by cancer cells. Furthermore, the flow of information in tumor cells may affect the expression of specific genes, facilitating immune system evasion and tumor dissemination and progression.^(^^30^^)^

## CONCLUSION

Any condition leading to or resulting from variations in gene expression can be studied in the light of miRNAs. Several studies have shown that miRNAs play an important role in carcinogenesis. Hence, variations in the profile of expression of these molecules can be used as markers for cancer identification and diagnosis, and may be explored as potential therapeutic targets. The role of miRNAs in gene expression control, and the fact that this interaction is cellular context-dependent, emphasize the applicability of these molecules as potential targets in personalized medicine. The release of miRNAs into the extracellular space and the circulation observed in body fluids suggest these molecules are important for intercellular communication, and may interfere with cancer dissemination and progression. The characteristics inherent to miRNAs and the wide availability of body fluid samples support the applicability of circulating miRNAs as biomarkers of disease progression and therapeutic response in some diseases, such as cancer. However, given the complexity of interactions with their targets and the dependency on cell´s characteristics, further studies are still needed before those molecules can be used in clinical practice.
