# Supplementary material for: MicroRNAs: understanding their role in gene expression and cancer
Source: Einstein (Sao Paulo). 2021 Jul 8;19:eRB5996. doi: 10.31744/einstein_journal/2021RB5996 (PMC8277234; doi:10.31744/einstein_journal/2021RB5996)
Supplement: Translation

# Translation: MicroRNAs: entendendo seu papel como reguladores da expressão gênica e seu envolvimento no câncer

**Authors:** Ariany Lima Jorge, Erik Ribeiro Pereira, Christian Sousa de Oliveira, Eduardo dos Santos Ferreira, Edmara Toledo Ninzoli Menon, Susana Nogueira Diniz, Julia Alejandra Pezuk

**Affiliations:** 1 São PauloSP Brasil Universidade Anhanguera de São Paulo, São Paulo, SP, Brasil.

**Keywords:** MicroRNAs, Expressão gênica, Neoplasias, Biomarcadores

## Abstract

Os microRNAs são pequenas moléculas de RNAs que regulam a expressão gênica das células. Com entre 17 e 25 nucleotídeos, essas pequenas moléculas reconhecem RNA mensageiro-alvo, por meio da complementariedade entre as sequências, e regulam sua tradução proteica. Todas as células humanas expressam diversos microRNAs. De fato, existem mais de 2.500 microRNAs descritos em humanos, relacionados com praticamente todos os processos biológicos. Devido ao seu papel como reguladores da expressão gênica, essas moléculas têm sido estudadas e relacionadas com algumas condições fisiológicas e patológicas específicas, sendo propostas como biomarcadores. Recentemente, foi descoberto que os microRNAs são normalmente liberados para fora da célula, onde participam da comunicação intercelular. MicroRNAs presentes nos fluidos biológicos são chamados de circulantes e têm sido encontrados em todos os fluidos corporais, porém o perfil de expressão é específico para cada tipo. O uso de microRNAs circulantes como marcadores biológicos apresenta vantagens relacionadas com a alta estabilidade dessas moléculas e a facilidade de obtenção de amostra. Adicionalmente, considerando que as alterações em microRNAs são dependentes das condições individuais, essas moléculas apresentam alto potencial de uso na medicina personalizada. De fato, a determinação do perfil de expressão de microRNAs pode auxiliar na identificação e diagnóstico de doenças, no monitoramento de respostas terapêuticas e na definição do prognóstico dos pacientes, auxiliando na escolha do tratamento. Nesta revisão são apresentados aspectos gerais dos microRNAs, e discute-se a importância dessas moléculas no câncer, visando a uma melhor compreensão de seu papel nessa doença.

## INTRODUÇÃO

Os microRNAs (miRNAs) são pequenas moléculas de ácido ribonucleico (RNA) que não codificam proteínas, os quais têm ganhado destaque nos últimos anos por seu papel como reguladores da expressão gênica e envolvimento em doenças, como as neoplasias. De fato, o câncer corresponde a um conjunto de doenças genéticas que podem ser causadas por inativação de genes supressores tumorais e/ou ativação de proto-oncogenes. A malignização celular implica no acúmulo de mutações no ácido desoxirribonucleico (DNA), que levam à desregulação da expressão gênica, a qual, por sua vez, acarreta consequências biológicas e funcionais para as células. Adicionalmente, alterações em processos epigenéticos também têm sido relacionadas com o desenvolvimento de tumores malignos. Dentre eles os miRNAs ganharam visibilidade devido a relação dessas moléculas com a aquisição de características malignas durante o processo de carcinogênese.^(^^1^^)^

Considerando esse contexto, aqui são apresentados e discutidos os avanços científicos dos últimos anos relacionados ao processo de geração e funcionamento de miRNAs, bem como sua relação com o câncer. Este artigo traz informações atualizadas sobre o papel dessas moléculas, discutindo a potencialidade de uso de miRNAs para o avanço no tratamento e no monitoramento do câncer.

## MicroRNAS

Os miRNAs foram descritos pela primeira vez na década de 1990 em *Caenorhabditis elegans,* e são atualmente considerados moléculas fundamentais na regulação da expressão gênica das células animais e vegetais.^(^^2^^)^Essas moléculas têm entre 17 e 25 nucleotídeos e regulam a expressão gênica de maneira pós-transcricional, alterando o padrão de tradução proteica por meio da interação com RNAs mensageiros (mRNAs).^(^^3^^)^Atualmente, existem mais de 18 mil miRNAs distintos em 168 espécies catalogados no banco de dados miRBase ( www.mirbase.org ). Na espécie humana, já foram identificados mais de 2.500 miRNAs, no entanto a função de muitos deles ainda não é totalmente compreendida.^(^^4^^)^Variações no padrão de expressão de miRNAs têm sido relacionadas com diversos processos biológicos e fisiológicos e descritas em praticamente todas as patologias humanas.^(^^5^^,^^6^^)^

## BIOGÊNESES DOS MicroRNAS

O processo de geração de miRNAs é complexo, inicia-se no núcleo e é finalizado no citoplasma, além de incluir a participação de diversas enzimas e complexos proteicos celulares, que regulam todo o percurso até a produção de miRNAs maduros capazes de desempenhar sua função ( Figura 1 ). Existem ao menos três vias de produção de miRNAs conhecidas, sendo a canônica a mais estudada.^(^^3^^)^

**Figura 1:** Biogênese canônica de microRNAs. Os microRNAs são primeiramente traduzidos no núcleo em moléculas denominadas pri-microRNAs. A partir de sequências de DNA, os pri-microRNAs são processados pela enzima nuclear Drosha, dando origem a uma molécula menor, chamada pré-microRNA. O pré-microRNA é exportado ao citoplasma, com a ajuda da exportina-5, no qual será processado pela enzima Dicer, gerando uma molécula de microRNA de fita dupla, que se acoplará ao complexo proteico silenciador induzido por RNA. O microRNA associado ao complexo silenciador induzido por RNA terá suas fitas separadas, dando origem a microRNAs maduros e capazes de se ligar aos RNAs mensageiros, inibindo sua tradução

A biogênese por via canônica começa com a transcrição do DNA, sendo que os miRNAs podem estar codificados em regiões intragênicas (principalmente regiões intrônicas) e intergênicas, tanto da fita sense quanto da fita antisense do DNA. A transcrição é realizada geralmente pela RNA polimerase II e, alter­nativamente, pela RNA polimerase III.^(^^7^^)^O primeiro transcrito, chamado miRNA primário ou pri-miRNA, tem uma estrutura de *hairpin* , ou grampo de cabelo, existindo uma parte na qual a molécula está pareada formando uma região de dsRNA (RNA de cadeia dupla) e com seus extremos protegidos por encapamento (CAP - *capped* ) e cauda poli-A.^(^^8^^)^O pri-miRNA é processado no núcleo pelo complexo enzimático formado pela RNase III Drosha e a proteína Pasha ou *DiGeorge Syndrome Critical Region 8 protein* (DGCR8), que tem capacidade de se ligar essa molécula de RNA de cadeia dupla e remover a cauda de poli A e o encapamento dos extremos da mesma. Esse processamento resulta em uma molécula de RNA de dupla fita com aproximadamente 70 nucleotídeos, denominada miRNA precursor ou pré-miRNA, que é exportada para o citoplasma com o auxílio das proteínas exportina-5 e Ran-GTP.^(^^9^^)^No citoplasma, a enzima Dicer continua com o processamento do miRNA, removendo a alça não pareada da molécula e dando origem a um miRNA de fita dupla de aproximadamente 22 nucleotídeos. Alternativamente, a proteína argonauta 2 (Ago2) pode participar da clivagem do pré-miRNA no citoplasma.^(^^10^^)^Esse miRNA de fita dupla é associado a um complexo proteico chamado complexo silenciador induzido por RNA (RISC), formado por diversas proteínas, sendo as proteínas Ago um dos fatores mais importantes, uma vez que são as responsáveis pela interação com as moléculas de RNAs.^(^^11^^)^O RISC causa a separação das duas fitas do miRNA, e, geralmente, uma delas é degradada, enquanto a outra, que corresponde ao miRNA maduro, fica exposta para poder parear com o mRNA-alvo.^(^^3^^,^^8^^)^

No processo de biogêneses de miRNAs por vias não canônicas, a produção de pré-miRNAs ocorre no núcleo, a partir de outras moléculas, como *short hairpin RNA* (shRNAs), miRtron ou m7G-pre-miRN, sendo que existem também variações em algumas das etapas subsequentes. A produção de pré-miRNA a partir de miRtron requer a participação do spliceossomo no núcleo. Por outro lado, o m7G-pre-miRN precisa da proteína exportina-1 para ser transportado para o citoplasma. Por último, a diferença entre o processamento canônico e o não canônico, envolvendo os shRNAs, se deve a participação das proteínas Ago2 no lugar da Dicer.^(^^3^^,^^12^^)^

## MECANISMO DE AÇÃO DOS MicroRNAS

Para cumprir sua função, o miRNA se liga por meio do reconhecimento de mRNAs-alvo, de acordo com a complementariedade de sequências, alterando o padrão de tradução em proteínas dessas mRNAs. Esse pareamento pode ser total ou parcial, e quanto maior a complementariedade das sequências, mais forte e douradora parece ser essa interação. Os miRNAs são considerados importantes reguladores da expressão gênica, e seu mecanismo é dinâmico e influenciado pela localização dessas moléculas e pelos mecanismos de interação com seu alvo.^(^^3^^)^

O mecanismo de ação dos miRNAs incluem diversas etapas com a participação de várias proteínas.^(^^12^^)^O miRNA se liga ao seu alvo principalmente na região 3’ não traduzida (UTR) e, alternativamente, a outras regiões, como a 5’UTR, ou até em regiões promotoras do mRN-alvo, o que determina a consequência biológica dessa interação. A maioria dos miRNAs apresenta efeito inibitório na expressão gênica, causado pelo impedimento da tradução proteica do mRNA-alvo. No entanto, quando o miRNA se liga à região promotora de um gene, seu efeito pode ser oposto, causando a ativação de sua expressão.^(^^13^^)^

A interação entre o miRNAs e o mRNA-alvo pode resultar na degradação do mRNA, por meio do processo de clivagem dessa molécula, causada por uma endonuclease presente no RISC, ao qual o miRNA está associado.^(^^14^^)^Inicialmente, foi descrito que, para que isso aconteça, deveria haver complementaridade total entre as moléculas, e, no caso de complementariedade parcial, ocorreria apenas uma inibição temporária da tradução. No entanto, estudos mais recentes têm demonstrado que, em células animais, a maioria dos miRNAs apresenta apenas complementariedade parcial ao mRNA-alvo, o que parece ser suficiente para inibir de maneira permanente sua tradução.^(^^5^^,^^15^^)^

O pequeno tamanho dos miRNAs e a tolerância de complementariedade parcial com seu alvo permitem a interação com grande número de mRNAs. Assim, as consequências biológicas de variações em um miRNA determinado podem ser diferentes, dependo do contexto celular, fazendo-se necessária a verificação experimental, uma vez que o efeito celular do miRNA é dependente da função do mRNA-alvo. Um único miRNA pode ter consequências biológicas opostas em células diferentes, no caso de apresentar complementariedade de sequências com mRNAs-alvo com funções antagônicas. Desse modo, para entender o papel de cada miRNA, é fundamental considerar o perfil de mRNA transcritos em cada tipo celular. A rede de interação miRNA-mRNA é complexa, e os modelos bioinformáticos podem auxiliar no entendimento dessa relação, para identificar efeitos aditivos ou contraditórios entre miRNAs distintos.^(^^3^^)^

Adicionalmente, cada mRNA pode ser alvo de mais de um miRNA, e, quando se pretende entender os efeitos de um miRNA determinado, é necessário ponderar a interação entre o mRNA-alvo e outros miRNAs, uma vez que o desfecho biológico pode ser dependente da adição de efeitos dos diversos miRNAs expressos na mesma célula.^(^^4^^)^Assim, é possível afirmar que um determinado miRNA pode regular processos opostos em distintos tipos celulares, como, por exemplo, aumentar a proliferação celular e da taxa de apoptose, ainda que níveis semelhantes de um miRNA específico podem não ter o mesmo efeito biológico, uma vez que dependem dos alvos e da interação com outras moléculas em um determinado tipo celular.

## MicroRNAS NO CÂNCER

O papel dos miRNAs no câncer vem sendo pesquisado desde o começo dos anos 2000, quando foi publicado o estudo realizado por Calin et al.^(^^16^^)^Eles mostraram que pacientes com leucemia linfoblástica crônica apresentam diminuição na expressão dos miR-15 e miR-16. A partir de então, alterações na expressão de diversos miRNAs em células tumorais têm apontando relação importante entre a desregulação dessas moléculas e o processo de carcinogênese.^(^^17^^,^^18^^)^Teoricamente, todos os tipos de tumores apresentam desregulação na expressão de algum tipo de miRNA. De fato, hoje sabemos que mais da metade dos genes que codificam miRNAs em humanos está localizada em regiões genômicas que foram descritas como desreguladas no câncer.^(^^4^^,^^19^^)^

Os miRNAs relacionados com o câncer podem ser classificados, de acordo com a função do mRNA-alvo, em supressores tumorais e miRNAs oncogênicos (oncomiRs). Essa segregação considera a habilidade dessas moléculas em interferir nos processos relacionados à carcinogênese, incluindo mecanismos associados com a migração e invasão celular, a apoptose e a proliferação. Pelo fato de a maioria dos miRNAs desempenhar um papel inibitório na expressão do mRNA-alvo, em geral sua classificação é oposta à de seu alvo. Assim, os miRNAs que agem como supressores tumorais são aqueles que regulam a expressão de mRNAs necessários para a divisão ou sobrevida celular, e os oncomiRs caracterizam-se por ter sua expressão aumentada nas células cancerosas, impedindo a atuação de genes capazes de suprimir o crescimento do tumor, estimulando a divisão de células cancerosa.^(^^1^^)^

As mudanças no padrão de expressão de miRNAs observadas nas células cancerosas demonstram a importância destas no surgimento e na progressão do câncer.^(^^20^^)^Variações no perfil de miRNAs têm sido associadas com processos que determinam a progressão do câncer, a formação de metástase e até com as taxas de morte celular, podendo ser relacionadas com o prognóstico do paciente. A desregulação na expressão de miRNAs observada em tumores malignos que progridem ou metastizam justifica a exploração dessas moléculas como potencial alvo terapêutico para neoplasias em estádio avançado.^(^^21^^)^Adicionalmente, a definição do perfil de expressão de miRNAs pode auxiliar na identificação e no diagnóstico de subtipos tumorais, podendo auxiliar a rever o prognóstico dos pacientes oncológicos.^(^^17^^,^^18^^)^

Nos últimos anos, a quantidade de estudos investigando a relação entre miRNAs e câncer tem crescido exponencialmente. Levantamento no PubMed^®^( www.pubmed.ncbi.nlm.nih.gov ) e na *Scientific Electronic Library Online* (SciELO) ( www.scielo.org ) das publicações científicas sobre o tema durante os últimos 7 anos demonstra o aumento no número de pesquisas científicas sobre miRNAs ( Tabela 1 ).

**Tabela 1:** Quantidade de artigos publicados por ano relacionando microRNAs e câncer, nos últimos 7 anos, de acordo com os descritores citados no título ou resumo

Base de dados	Descritores	2013	2014	2015	2016	2017	2018	2019	Total
PubMed^®^	“miRNA” e “câncer”	1.387	1.680	1.988	1.980	2.123	2.454	2.737	14.349
	“microRNA” e “câncer”	1.579	1.976	2.361	2.402	2.775	2.949	3.222	17.264
	“miRNA circulante” e “câncer”	24	32	33	32	35	35	49	240
	“microRNA circulante” e “câncer”	19	26	40	37	35	32	37	226
SciELO	“miRNA e “câncer”	4	2	1	4	4	4	3	22
	“microRNA e “câncer”	2	0	1	2	6	4	2	17
	“miRNA circulantes” e “câncer”	0	0	1	0	0	0	0	1
	“microRNA circulante” e “câncer”	0	0	1	0	0	0	0	1

Entender as particularidades de cada miRNA é fundamental para compreender o papel que essas moléculas têm nas doenças e no câncer. Nesse sentido, é possível encontrar diversas bases de que coletam e disponibilizam informações específicas para cada miRNAs. Com o aumento de informações científicas sobre miRNAs, diversas bases de dados têm sido criadas para registrar características sobre tais moléculas. No *site* da tools4miRs ( www.tools4mirs.org ), são referenciadas diversas páginas *web* que coletam informações sobre miRNAs, nas quais podem ser encontradas as sequências, os mRNAs-alvo e os estudos científicos publicados sobre cada miRNA.

Uma das bases de dados mais usadas é a miRBase. Nela são explicitadas as propriedades de cada miRNA, incluindo nomes atuais e anteriores, publicações relacionadas e a própria sequência da molécula. Nesse *site* , são ainda mencionados diversos *links* para outras bases de dados com informações complementares para cada miRNA.^(^^4^^)^

No *site* miRmine, pode ser encontrada a descrição do nível de expressão de miRNAs por tipo de tecido ou linhagem celular ( https://guanfiles.dcmb.med.umich.edu/mirmine/ ), o que facilita o estudo de alterações patológicas potencialmente exploradas como biomarcadores. Adicionalmente, dados de expressão de miRNAs especificamente em células cancerosas podem ser verificados nas bases de dados miRCancer ( http://mircancer.ecu.edu/ ), que disponibiliza o nível de expressão de acordo com o tipo de neoplasia usando dados científicos publicados.

Ainda, para entender o efeito biológico da expressão de um determinado miRNA, podem ser consultados *sites* e *softwares* , que, por meio de algoritmos matemáticos e análises de complementariedade de sequência entre as moléculas, preveem quais são os potenciais mRNAs-alvo para cada miRNA maduro. Dentre eles, destacam-se o miRDB ( http://mirdb.org/ ) e o miRTar ( https://mcube.nju.edu.cn/jwang/mirTar/docs/mirTar/ ). Cada *software* usa de algoritmos específicos, e os resultados podem variar entre os *sites* utilizados, sendo fundamental uma análise crítica dos resultados. Complementarmente, a esses dados, ainda existem sítios na internet que registram apenas alvos validados experimentalmente para cada miRNA, estando entre os mais divulgados para essa categoria o miRTarBase ( https://mirtarbase.cuhk.edu.cn/∼miRTarBase/miRTarBase_2019/php/index.php ) e o TarBase ( https://carolina.imis.athena-innovation.gr ).

A maioria das informações disponíveis nesses sites e softwares é atualizada periodicamente com dados provenientes de pesquisas científicas publicadas. Novas bases de dados são criadas e reformuladas constantemente para incluir as informações atualizadas, de acordo com os conhecimentos adquiridos e publicados sobre miRNAs.

## MicroRNAS CIRCULANTES

Recentemente, tem sido demonstrado que os miRNAs não se findam apenas ao interior celular. Essas moléculas também são liberadas para fora das células, podendo ser encontradas circulando em diversos fluidos corporais, sendo, nesses casos, chamadas de miRNAs circulantes (c-miRNAs).^(^^20^^)^Os c-miRNAs parecem desempenhar papel importante na comunicação intercelular, executando uma função tanto em processos fisiológicos como em patológicos. Os mecanismos de transporte e liberação de c-miRNAs implicam na participação e na associação dessas moléculas com proteínas e/ou o encapsulamento em vesículas formadas por lipoproteínas, que as protegem da degradação enzimática. Porém os mecanismos que controlam sua liberação ainda não foram totalmente desvendados.^(^^22^^,^^23^^)^

A primeira publicação que relata a detecção de c-miRNAs é de 2008, quando foi descrita a presença de miR-155 e miR-21 em quantidades diferentes no soro de pessoas saudáveis quando comparadas com pacientes com linfoma de células B.^(^^24^^)^Logo em seguida, foi demonstrada a presença de c-miRNAs em diferentes quantidades em outros tipos de líquidos biológicos, incluindo fluido seminal, colostro, urina, saliva, leite materno, lágrimas, líquido amniótico, secreção brônquica, plasma, líquido pleural, líquido peritoneal e líquido cefalorraquidiano.^(^^22^^)^A presença de c-miRNAs nos diversos líquidos biológicos permite que essas moléculas tenham capacidade de transferência horizontal, passando entre diferentes tecidos e desempenhando função pleiotrópica, uma vez que podem causar efeitos biológicos a curtas e longas distâncias.

O mecanismo de seleção e liberação de c-miRNAs é controlado e implica em perfil específico em cada tipo fluido biológico, sendo que alterações em células distintas acarretam em variações particulares, que podem ser exploradas como marcadores biológicos de condições fisiológicas ou patológicas.^(^^3^^)^De fato, tem sido sugerido usar o perfil de c-miRNAs no sangue, no plasma, no soro ou em outros fluidos corporais, para identificar ou monitorar a evolução de doenças e as respostas terapêuticas.^(^^22^^)^As principais vantagens do uso de c-miRNAs são alta especificidade e sensibilidade dessas moléculas; facilidade de obtenção de amostras e alta estabilidade. Isso facilita sua manipulação e favorece sua exploração como ferramenta importante para a medicina personalizada. No entanto, a falta de protocolos padronizados ainda limita seu uso na prática clínica, principalmente pela falta de normalizadores universais que permitam a quantificação de maneira que os resultados possam ser comparados.^(^^21^^,^^25^^,^^26^^)^

### miRNAs circulantes no câncer

As alterações biológicas celulares observadas durante o processo de carcinogênese podem acarretar variação no perfil de c-miRNAs de maneira específica. Porém tem sido reportado que mudanças na expressão de miRNAs intracelulares nem sempre se correlacionam diretamente com as variações na expressão de c-miRNAs observadas em fluidos biológicos. De fato, para entender essa relação, é necessário considerar os efeitos que o crescimento de um tumor tem no organismo. As modificações no perfil de c-miRNA podem ser ocasionadas não apenas pelo aumento ou pela alteração, observados nas células cancerosa, mas também podem ser conse­quências das respostas do sistema imune e/ou de processos inflamatórios, ou, ainda, serem causada pela terapia ou por mudanças fisiológicas.^(^^18^^,^^21^^)^

No entanto, nos últimos anos, a determinação do perfil c-miRNAs tem sido proposta para identificar, prever o prognóstico e acompanhar a evolução de diversas neoplasias.^(^^21^^)^O reconhecimento das variações no perfil de c-miRNAs é promissor, principalmente quando são levadas em consideração as vantagens de usar essas moléculas na classificação dos tumores.^(^^27^^,^^28^^)^Adicionalmente, variações do perfil de c-miRNAs permitem identificar precocemente a resposta terapêutica, podendo ser usadas para predizer resultados clínicos e inferir as taxas de sobrevivência.^(^^29^^)^

Considerando que os c-miRNAs tem um papel na comunicação intercelular, essas moléculas podem afetar a expressão gênica de células distantes e, dessa forma, desempenhar outro papel importante, dessa vez nos processos de progressão tumoral e de formação de metástases.^(^^29^^)^De fato, acredita-se que os c-miRNAs participam da preparação do nicho de metástase, facilitando a colonização de novos ambientes pelas células cancerígenas. Adicionalmente, esse fluxo de informação das células tumorais poderia ainda se importante no processo de evasão do sistema imunológico, pela regulação na expressão de genes específicos, favorecendo a disseminação e a progressão tumoral.^(^^30^^)^

## CONCLUSÃO

Qualquer condição que cause ou seja causada por variações na expressão gênica pode ser estudada à luz dos miRNAs. Diversos estudos comprovaram que os miRNAs são importantes moléculas no processo de car­cinogênese. Por isso, variações no perfil de expressão dessas moléculas podem ser utilizadas como marcadores de identificação e diagnóstico do câncer e, ainda, exploradas como potenciais alvos terapêuticos. As funções que os miRNAs desempenham no controle da expressão gênica e o fato de a interação ser dependente do contexto celular individual destacam essas moléculas como potenciais alvos da medicina personalizada. Adicionalmente, a liberação de miRNAs para o espaço extracelular e a circulação observada nos fluidos biológicos apontam a importância dessas moléculas na comunicação intercelular, sugerindo que podem ter um papel durante a progressão e a disseminação do câncer. As características intrínsecas dos miRNAs, junto da facilidade de obtenção de amostra de fluidos corporais, destacam os miRNAs circulantes como biomarcadores para o acompanhamento da evolução e da resposta terapêutica de patologias como o câncer. No entanto devido à complexidade de interação com os alvos e a dependência com as características celulares, ainda são necessários mais estudos para poder utilizar essas moléculas na prática clínica.
